# Reconciliation feasibility in the presence of gene duplication, loss, and coalescence with multiple individuals per species

**DOI:** 10.1186/s12859-017-1701-1

**Published:** 2017-06-05

**Authors:** Jennifer Rogers, Andrew Fishberg, Nora Youngs, Yi-Chieh Wu

**Affiliations:** 1 Department of Computer Science, Harvey Mudd College, Claremont, 91711 California USA; 2 Department of Mathematics, Harvey Mudd College, Claremont, 91711 California USA; 3 Current Address: Department of Mathematics and Statistics, Colby College, Waterville, 04901 Maine USA

**Keywords:** Phylogenetics, Reconciliation, Coalescence, Incomplete lineage sorting, Gene duplication and loss

## Abstract

**Background:**

In phylogenetics, we often seek to reconcile gene trees with species trees within the framework of an evolutionary model. While the most popular models for eukaryotic species allow for only gene duplication and gene loss or only multispecies coalescence, recent work has combined these phenomena through a reconciliation structure, the labeled coalescent tree (LCT), that simultaneously describes the duplication-loss and coalescent history of a gene family. However, the LCT makes the simplifying assumption that only one individual is sampled per species whereas, with advances in gene sequencing, we now have access to multiple samples per species.

**Results:**

We demonstrate that with these additional samples, there exist gene tree topologies that are impossible to reconcile with any species tree. In particular, the multiple samples enforce new constraints on the placement of duplications within a valid reconciliation. To model these constraints, we extend the LCT to a new structure, the partially labeled coalescent tree (PLCT) and demonstrate how to use the PLCT to evaluate the feasibility of a gene tree topology. We apply our algorithm to two clades of apes and flies to characterize possible sources of infeasibility.

**Conclusion:**

Going forward, we believe that this model represents a first step towards understanding reconciliations in duplication-loss-coalescence models with multiple samples per species.

**Electronic supplementary material:**

The online version of this article (doi:10.1186/s12859-017-1701-1) contains supplementary material, which is available to authorized users.

## Background

In evolutionary biology, a phylogenetic tree describes evolutionary relationships among a collection of taxonomic units, for example, genes or species. To understand the evolutionary history of a *gene family*, or a set of genes with detectable shared ancestry, we rely on two types of phylogenetic trees: the *species tree* that describes how a set of species have speciated, and the *gene tree* that describes how a set of genes sampled from these species have diverged. The gene tree can be thought of as evolving “inside” the species tree, and this nesting is represented as a *reconciliation* that indicates the particular number and order of evolutionary events that gave rise to the gene tree.

When the gene tree and species tree are congruent, the gene tree topology can be explained through speciation events alone. However, when the two trees are incongruent, we must account for the differences by postulating additional evolutionary events. For example, the number of loci per species could change due to gene duplication and loss [1–6] or additionally horizontal gene transfer [3, 7–11]. Stochastic fixation of polymorphisms in a population could result in incomplete lineage sorting [3, 12–17]. There could be events in the species history not represented in a species tree such as hybridization [18–21]. Or convergent evolution, in which similar traits evolved independently rather than as a result of shared ancestry, may have occurred, for example through gene conversion [22–25].

**Fig. 1:**
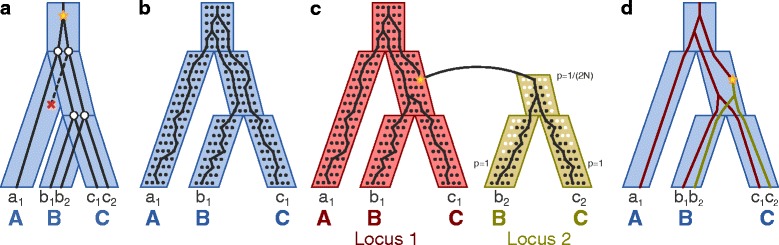
Evolutionary models and reconciliation structures. **a** In the duplication-loss model, incongruence between the gene tree (*black*) and species tree (*blue*) can be explained using gene duplications (*yellow star*) and gene losses (*red* “x”). **b** In a multispecies coalescent model, incongruence between the gene tree and species tree can be explained due to incomplete lineage sorting (ILS). **c** The unified model proposed by Rasmussen and Kellis [36] combines the duplication-loss and multispecies coalescent models. For an alternative view of this model, in which the traditional duplication-loss and coalescent processes are decoupled, see Additional file 1: Figure S1. **d** The LCT combines the species tree, locus tree, gene tree, and reconciliations between them into a single structure. [Figure and caption adapted with permission from Wu et al. [37] and Rasmussen and Kellis [36]]

In this work, we focus on two of the most popular evolutionary models for eukaryotic organisms: the *duplication-loss model* that allows for gene duplications and gene losses (Fig. 1 a) and the *multispecies coalescent model* that allows for incomplete lineage sorting (Fig. 1 b). Many reconciliation methods have been developed that focus on only one of these models. For inferring duplications and losses, reconciliation can be performed using a parsimony [1, 2, 26–28] or probabilistic [4, 6, 29] framework. For inferring evolution in the presence of incomplete lineage sorting, similar parsimony [3, 30] and probabilistic [15] methods exist, though these are often used to estimate ancestral population sizes or divergence times [15], to reconstruct species trees [31, 32], or both [33, 34].

Only a few methods consider reconciliation under a *duplication-loss-coalescence, or DLC, model*, which, as its name implies, allows for duplication, loss, *and* coalescence. For example, NOTUNG [35] reconciles gene trees against a non-binary species tree to minimize the duplication-loss cost while allowing for possible deep coalescence at unresolved nodes in the species tree. While this parsimony framework is simple, it cannot capture all possible evolutionary histories. More recent algorithms have relied on a unified generative model, DLCoal, that introduces an intermediate *locus tree* (Fig. 1 c; [36]). Under this model, the gene tree (also known as the *coalescent tree*) evolves within the locus tree according to a multispecies coalescent model, and the locus tree evolves within the species tree according to a duplication-loss model. Subsequently, a new reconciliation structure, the labeled coalescent tree (LCT), was introduced that simultaneously describes the three trees and reconciliations between them (Fig. 1 d; [37]). The associated reconciliation algorithms DLCoalRecon and DLCpar infer the maximum *a posteriori* or a most parsimonious reconciliation, respectively, and substantially improve homolog and event inference.

However, both DLCoalRecon and DLCpar assume that each extant species is represented by a single haploid sample. But as more genomes are sequenced and variants genotyped, it will become increasingly important to incorporate the additional information provided by the multiple samples into phylogenetic algorithms. Here, we consider for the first time the problem of gene tree-species tree reconciliation under a DLC model with multiple samples per species. However, rather than present a full reconciliation method, we consider the subproblem of reconciliation feasibility. That is, previously, DLCoalRecon and DLCpar relied on the fact that, regardless of the gene tree topology, a feasible reconciliation always exists. The proof is trivial: any such gene tree (with one haploid sample but possibly multiple loci per species) can be reconciled against any species tree under a duplication-loss model alone [38]. Of course, not all reconciliations are parsimonious or probable; nevertheless, the existence of a universal reconciliation strategy allows research to focus on finding an optimal one.

In contrast, when using multiple haploid samples per species, we can no longer assume that a feasible reconciliation exists. For this problem, we present several contributions:

- We demonstrate that, with multiple haploid samples per species, if at least one species contains multiple loci, then regardless of the species tree topology, there exist gene tree topologies for which no valid reconciliation is possible.
- We present an algorithm for determining reconciliation feasibility. Our algorithm relies on a new reconciliation structure, the partially labeled coalescent tree (PLCT), to capture the constraints implied by the multiple loci and multiple samples. In brief, the PLCT is a gene tree in which each branch is labeled with the locus in which it evolved, and the labeling is partial because not all branches necessarily have labels and because multiple labels may correspond to the same locus. We further introduce the locus equivalence graph (LEG) to capture the constraints among loci within the PLCT and demonstrate how connected components within the LEG can be used to determine reconciliation feasibility.

To demonstrate the utility of our approach, we have applied it to both a real primate and a simulated fly data set to characterize the percentage of feasible and infeasible gene trees and understand how various user choices and data set metrics, such as the gene tree reconstruction algorithm, the number of samples, and the level of branch support and ILS, affect feasibility. The PLCT software and data are freely available for download at http://www.cs.hmc.edu/~yjw/software/plct.

## Methods

### Gene family evolution under duplication, loss, and coalescence

To understand how gene families evolve through gene duplication, gene loss, and coalescence, we start by reviewing the DLCoal model that combines the duplication-loss and multispecies coalescent models [36]. The DLCoal model makes the following assumptions:

1. Any incongruence between the gene tree and species tree can be explained through duplication, loss, and coalescence. Furthermore, each duplication creates a unique new locus that is unlinked with the original locus, allowing coalescence within the original and new loci to occur independently, and there is no gene conversion between duplicated loci.
2. Duplication and loss events do not fix differently in descendant species; that is, they do not undergo hemiplasy (Additional file 1: Figure S2; [39]). Equivalently, all duplications and losses either always go extinct (*p*=0) or fix (*p*=1) in all descendant lineages, allowing us to separate the duplication-loss process from the coalescent process.
3. Each extant species is represented by a single haploid sample; that is, within each gene family, multiple genes from the same extant species are sampled from multiple loci in a single individual as opposed to being sampled from the same locus across multiple individuals.

Assumption 1 is applicable to evolution within eukaryotic species, and assumption 2 was shown to affect only a small number of gene trees in simulation with biologically realistic parameters [36]. We relax assumption 3 in this work.

We now consider the gene family illustrated in Fig. 1 c. In this example, a duplication occurs in one chromosome along the branch ancestral to species *B* and *C*, creating a new locus (“locus 2”) in the genome distinct from the original locus (“locus 1”). At the new locus, this duplicate evolves within the population according to the Wright-Fisher process [12, 14–16, 40] until it eventually fixates. Thus, the sampled genomes of *A*, *B*, and *C* contain genes *a*_1_, *b*_1_, *b*_2_, *c*_1_, and *c*_2_, and their phylogenetic tree is a “traceback” in the combined Wright-Fisher processes of loci 1 and 2. Furthermore, the red and yellow trees representing loci 1 and 2 form an intermediate *locus tree* that is distinct from the gene tree and species tree and describes how loci are created and destroyed. To disentangle the effects of duplication-loss and coalescence, we can think of the gene tree as evolving “inside” the locus tree, with a multispecies coalescent process within each locus, and we can think of the locus tree as evolving “inside” the species tree according to a duplication-loss process (Additional file 1: Figure S1). As the gene tree of this model represents the history of gene sequences as they coalesce within the locus tree, we will use the term coalescent tree and gene tree interchangeably throughout the remainder of this manuscript.

### Reconciliation using the labeled coalescent tree

In the DLCoal model, evolutionary history is captured through three trees and two reconciliations: the gene, locus, and species trees, and the gene tree-locus tree and locus tree-species tree reconciliations. The labeled coalescent tree (LCT) combines this history into a single reconciliation structure (Fig. 1 d; [37]). As a full description of the LCT is not necessary for our purposes, we focus on the concepts essential to our reconciliation feasibility algorithm. First, duplications occur along branches in the LCT. In contrast to duplications at nodes of the locus tree, duplications in the LCT denote that the locus has changed at some point along the branch. By placing duplications along branches, we can capture the delay between a duplication event and the time at which the lineage with the duplicate coalesces with another lineage in the original locus. For example, in the scenario of Fig. 1 d, a duplication occurs in the ancestor of species *B* and *C*, but the lineage with the duplicate coalesces with a lineage in the original locus in the root species. Second, the LCT labels each node and branch with the locus in which the gene evolves; for branches with a duplication, one side of the branch (before the duplication) is labeled with the original locus and the other side (after the duplication) with the new locus.

Let us consider one version of the reconciliation problem in which we are given a gene tree, a species tree, and a leaf mapping that, for each extant gene, defines the extant species from which it was sampled. Both trees are full, rooted, and binary, and the leaf mapping indicates only the species to which each extant gene belongs. In particular, we have no knowledge of how loci across different species are related. For this problem, if each species is represented by a single haploid sample, then regardless of the gene tree topology, a valid reconciliation exists.

### Constraints introduced by multiple samples

We now extend our reconciliation problem to consider the case in which at least one species is sequenced from multiple haploid samples (requiring a coalescence-aware model) and at multiple loci (requiring a duplication-aware model). We assume that we know the species-specific locus from which each gene is sampled, as would be the case when variants are mapped onto a reference genome. But as before, we have no knowledge of how loci across different species are related. Furthermore, there may exist copy number variation across the samples in that different samples from the same species contain different loci.

**Fig. 2:**
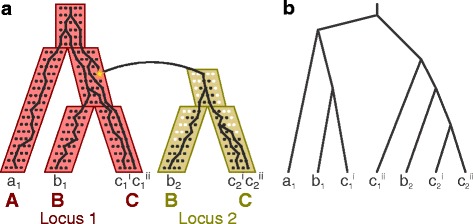
Multiple samples. **a** The unified model allows for multiple samples per species (sampled individuals denoted in superscript). **b** A reconstructed gene tree shows different histories for the multiple samples, and a reconciliation must simultaneously explain these histories

To demonstrate how multiple samples might provide additional information for the reconciliation problem, consider the gene family illustrated in Fig. 2 a. While the duplication-loss history of this family is identical to that of Fig. 1 c, we now have access to two individuals (the original sample *i* and a new sample *ii*) from species *C*. Furthermore, the coalescent histories of these samples differ. In particular, the gene tree supports two placements for gene *c*_1_ with respect to the other genes. (In contrast, because the two samples for gene *c*_2_ coalesce with each other before coalescing with other lineages, the additional sample provides no additional information about this gene.) Given only a reconstructed gene tree (Fig. 2 b), a reconciliation must simultaneously explain the history of all samples.

**Fig. 3:**
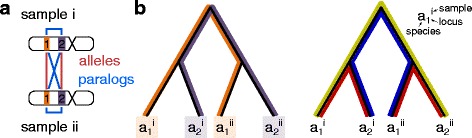
Allele and paralog constraints. **a** Genes are sampled at two loci 1 and 2 from two individuals *i* and *ii* in a single species *A*. Within this species, genes at the same locus (across multiple individuals) must be alleles, and genes at different loci (regardless of individual) must be paralogs. **b** A valid reconciliation must not include a duplication along the path between alleles (*left*, each color corresponds to one locus, and no colored branch can have a duplication). At the same time, a valid reconciliation must include a duplication along the path between paralogs (*right*, every colored path must have a duplication on at least one branch of the path). There is no way to simultaneously satisfy these constraints, so this gene tree is not reconcilable

But under these assumptions, the reconciliation is no longer trivially feasible because the multiple samples introduce *allele constraints* and the multiple loci introduce *paralog constraints*. That is, within a species, genes at the same locus across multiple samples must be alleles, and genes at different loci must be paralogs. As an example of how allele and paralog constraints may conflict, consider sampling two genes (from locus 1 and locus 2) in two individuals (samples *i* and *ii*) from a single species *A* (Fig. 3 a). The reconstructed gene tree (Fig. 3 b) suggests that the genes within an individual are more closely related than the same gene across multiple individuals; such a gene tree may have been the result of noisy gene sequencing or reconstruction error, or due to violations of our model assumptions, for example, through gene conversion within each individual.

We now demonstrate that this gene tree is infeasible under a DLC model. Genes $a_{1}^{i}$ and $a_{1}^{ii}$ are from the same locus 1, so they must be alleles, and a valid reconciliation must not have any duplication along the path between these two leaves (Fig. 3 b, orange). Similarly, genes $a_{2}^{i}$ and $a_{2}^{ii}$ from locus 2 must be alleles, further constraining the location of duplications (Fig. 3 b, purple). Next, locus 1 and locus 2 are distinct loci within the same species; therefore, any pair of genes, one from locus 1 and one from locus 2, must be paralogs, and a valid reconciliation must have at least one duplication along the path between each gene pair (Fig. 3 b, right). Now suppose that we wanted to add a duplication between paralogs $a_{1}^{i}$ and $a_{2}^{i}$. There is no place to put this duplication because we have already prohibited duplications on every branch between $a_{1}^{i}$ and $a_{2}^{i}$. Thus, there is no way to simultaneously satisfy these allele and paralog constraints, and the gene tree in this example is not reconcilable.

### An algorithm to determine reconciliation feasibility

We have seen that, in the presence of multiple samples and loci per species, not all gene trees are reconcilable. Furthermore, whether a gene tree is reconcilable depends only on allele and paralog constraints, which in turn depend on the gene tree topology and the leaf mapping but are independent of the species tree and of the rooting of the gene tree. Thus, while we use the term reconciliation feasibility throughout this manuscript, a more appropriate term might be *gene tree feasibility* under a reconciliation model.

**Fig. 4:**
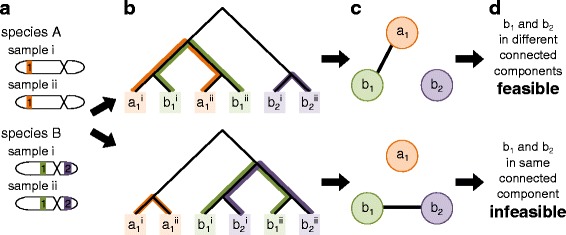
Reconciliation feasibility. **a** The sampled species, loci, and individuals. We assume knowledge of the species-specific locus from which each gene is sampled. **b** For a gene tree (*black*), the PLCT uses alleles to label branches along which no duplications are allowed (*colored lines*). **c** The LEG contains one node per species-specific locus and encodes overlapping labels in the PLCT as edges in the LEG. **d** A gene tree has a feasible reconciliation if and only if every connected component of the LEG contains no more than one locus from each species

We now present an algorithm to determine whether a gene tree is reconcilable given the constraints imposed by the inclusion of multiple samples and multiple loci (Fig. 4). Our algorithm consists of two new structures: the *partially labeled coalescent tree (PLCT)* that describes the constraints on the placement of duplications in the gene tree, and the *locus equivalence graph (LEG)* that describes the set of loci that must be orthologous. To determine feasibility, we examine the pairs of loci within the LEG that must be paralogs. If any pair of loci is constrained to be both orthologs and paralogs, then we conclude that the gene tree has no valid reconciliation. We describe these steps in more detail below. Here, we focus on the algorithmic intuition. Technical details, including pseudocode, a formal proof of correctness, an analysis of time complexity, and optimizations, are provided in Additional file 1: Section S1.

#### Generating the partially labeled coalescent tree

We are given as input a full, binary gene tree and a leaf mapping that, for each extant gene, defines the extant species from which it was sampled and the species-specific locus at which it was sampled (Fig. 4 a). Our goal is to label the gene tree branches along which duplications cannot have occurred.

To construct these constraints, we consider each set of genes mapped to the same species and locus. Each pair of genes within this set must be alleles, so duplications cannot have occurred along the path between any pair. To denote this constraint, we label the branches along these paths with a unique color corresponding to the species-specific locus (Fig. 4 b). We then repeat this process for each locus of each species. Thus, a branch of the PLCT may be labeled with multiple colors if it is constrained by multiple species-specific loci.

#### Generating the locus equivalence graph

Given a PLCT, our goal is to encode the set of species-specific loci that must be orthologous. However, rather than consider orthologs, we will consider the stronger concept of locus equivalency. Two loci in different species are *equivalent* if they derived from their most recent common ancestor through speciation events alone. As an example, in the scenario of Fig. 1 c, each species has its own species-specific “locus 1” (*a*_1_, *b*_1_, *c*_1_) that derived from the original “locus 1” in the root species through speciations. Note that equivalent loci must be orthologous but orthologous loci may not be equivalent as duplications could have occurred since the common ancestor. Furthermore, because only speciations are allowed, locus equivalency is a transitive relationship.

To encode locus equivalencies, we construct a graph in which nodes denote species-specific loci and edges denote the equivalency constraint. We start by creating a graph with one vertex for each species-specific locus. Next, for every branch of the PLCT with multiple labels, we add an edge to the LEG between all pairwise combinations of these labels (Fig. 4 c). To understand the rationale, recall that each label in the PLCT corresponds to one species-specific locus and that the PLCT can assign multiple labels to each gene tree branch. Since each branch also corresponds to a gene lineage at a specific point in time and this lineage must exist at only one locus, if a branch has multiple labels, these labels must correspond to the same locus and be equivalent.

#### Determining reconciliation feasibility

Finally, given a LEG, our goal is to determine whether the original input gene tree has a feasible reconciliation. We call a LEG reconcilable if and only if every connected component of the LEG contains no more than one locus from any species, and we claim that a gene tree is reconcilable if and only if its LEG is reconcilable.

First we show that if the LEG is irreconcilable, that is, if any connected component of the LEG contains multiple loci from a single species, then the gene tree is irreconcilable. Since locus equivalency is transitive and implies orthology, each connected component of the LEG represents a set of species-specific loci that must be equivalent, and every pair within this set must be orthologs. However, we also know that distinct loci within the same species must be paralogs. Therefore, if any connected component of the LEG contains multiple loci from a single species, then a pair of genes is constrained to be both orthologs and paralogs. As no reconciliation can satisfy both constraints, the gene tree must be irreconcilable (Fig. 4 d, bottom).

Next we show that if the LEG is reconcilable, that is, every connected component of the LEG contains no more than one locus from a single species, then the gene tree is reconcilable. Note that if we required that loci within the same connected component of the LEG be equivalent and loci across different connected components be non-equivalent, then such a reconciliation is valid (Fig. 4 d, top). We can induce the former constraint by restricting duplications from occurring on any labeled branch of the PLCT. Similarly, we can induce the latter constraint by inserting duplications on unlabeled branches between nodes in different connected components of the LEG. We emphasize that this reconciliation, though valid, may not be parsimonious nor probable.

Lastly, we comment briefly on our algorithm as applied to non-binary gene trees. In such cases, if the LEG is irreconcilable, then the gene tree is irreconcilable. However, if the LEG is reconcilable, then the reconciliation feasibility of the gene tree is unknown (Additional file 1: Theorem S1.1).

## Results and discussion

### Biological data set of ape genomes

To assess our algorithm on a real data set, we analyzed 6298 gene families across seven species or subspecies of great apes, with data obtained from Prado-Martinez et al. [41] and Flicek et al. [42] and trees reconstructed using maximum parsimony (PHYLIP [43]), neighbor-joining (BioNJ [44]), and maximum likelihood (PhyML [45], RAxML [46]) (Additional file 1: Section S2, Tab 1).

**Table 1:** Reconciliation infeasibility in apes data set

Phylogenetic program	Trees ^a^	Loci ^b^	Monophyletic trees ^c^	Monophyletic loci ^d^	Infeasible ^e^
PHYLIP	76	1394	3 (3.9%)	1115 (80.0%)	35 40.8
BioNJ	6298	125,928	5 (0.1%)	63,013 (50.0%)	366 5.8
PhyML	6297	125,914	46 (0.7%)	80,805 (64.2%)	229 3.6
RAxML	6298	125,928	34 (0.5%)	73,576 (58.4%)	213 3.4

^a^The number of gene trees considered for each program. For PHYLIP, since many trees were non-binary, we considered only trees for which we can definitively determine their reconciliation feasibility or infeasibility. In particular, non-binary trees with a reconcilable LEG were not considered. For PHYLIP and PhyML, one tree could not be reconstructed

^b^The number of species-specific loci across all trees

^c^The number and percentage of trees for which, for every species-specific loci, the genes in that loci were inferred to be monophyletic

^d^The number and percentage of species-specific loci for which the genes in that loci were inferred to be monophyletic

^e^The number and percentage of trees with infeasible reconciliations

To understand whether multiple samples add information to the gene tree, we investigated the monophyly of genes sampled at the same species and same locus. If such genes are inferred to be monophyletic, then the multiple samples agree on their relationship relative to other genes and contribute no added information over a single sample. We call a species-specific loci monophyletic if the genes within the locus are monophyletic, and we call a tree monophyletic if all loci within the tree are monophyletic. We find that 57.6% of loci and 0.46% of trees are monophyletic, suggesting some disagreement among the samples.

Additionally, we find that despite the low percentage of monophyletic trees, 4.4% of trees are infeasible. We believe that many trees are feasible because most loci are monophyletic; therefore, only a few gene tree branches have multiple labels, resulting in less possibility for conflict in the LEG. For example, for RAxML gene trees, only 26.5% of branches are labeled and only 17.7% have multiple labels. While we have only investigated a few gene tree reconstruction algorithms, we find that the percentage of infeasible trees increases as reconstruction accuracy decreases, with maximum likelihood methods outperforming neighbor-joining methods [6, 47] and neighbor-joining methods outperforming parsimony methods [48, 49].

**Fig. 5:**
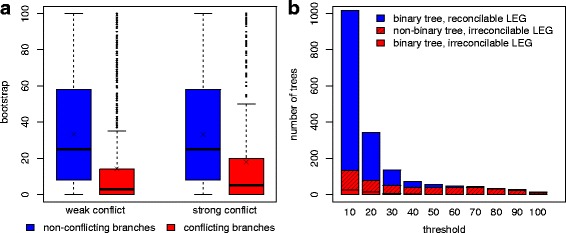
Reconciliation infeasibility due to poorly supported branches. **a** Bootstrap support among conflicting and non-conflicting branches. A branch is said to conflict if its labels are part of a connected component that contains multiple loci from a single species (*weak* conflict) or if its labels map to multiple loci from a single species (*strong* conflict). For both types of conflict, the distribution of bootstrap support for conflicting branches was significantly lower than the distribution for non-conflicting branches (mean denoted as ‘ ×’, statistics in Additional file 1: Table S1). **b** Reconciliation infeasibility after collapsing poorly supported branches. For each gene tree, we collapsed branches with bootstrap support below the threshold, generated LEGs for the resulting multifurcating gene trees, and evaluated the feasibility of the LEGs. Non-binary trees with a reconcilable LEG have unknown reconciliation feasibility and are not shown but constitute the remainder of the 6298 gene trees. As the threshold increases, the numbers of feasible (*blue*) and infeasible (*red*) gene trees decrease while the number of trees with unknown feasibility increases

Next, we hypothesized that reconciliation infeasibility was the result of poorly supported branches in the gene tree. To investigate this possible effect, we analyzed RAxML gene trees in two ways. In our first approach, recall that a conflict occurs in the LEG if any connected component contains multiple loci from a single species. After separating branches based on whether the branch labels are part of a conflicting connected component, we find that conflicting branches have significantly lower bootstrap support than non-conflicting branches (Fig. 5 a). This trend remains even after strengthening our definition of conflict to include only branches whose labels map to multiple loci from a single species. That is, the labels must directly conflict, which is equivalent to looking only for conflicts in neighboring nodes of the LEG rather than among the nodes in each connected component. In our second approach, we collapsed branches with bootstrap support below a threshold and evaluated the feasibility of the resulting gene trees (Fig. 5 b). Here, recall that non-binary gene trees with a reconcilable LEG have unknown reconciliation feasibility. As the threshold increases, the number of gene trees with such indeterminate feasibility increases. At the same time, the number of infeasible gene trees decreases, an expected result as fewer branches have conflicting labels, resulting in fewer conflicts in the LEG. And the number of feasible gene trees also decreases until eventually, a smaller percentage of gene trees are feasible than infeasible. Altogether, these results demonstrate that while reconciliation feasibility is affected by poorly supported branches, even robust gene trees with well-supported branches can be infeasible.

### Simulated data set

To evaluate our algorithm on a different clade, we used the simulated data set of twelve *Drosophila* previously developed by Rasmussen and Kellis [36] for evaluating reconciliations under a DLC model, supplemented to simulate multiple individuals per species and gene tree reconstruction error (Additional file 1: Section S3). In brief, we used a known species tree and parameters and simulated evolution with varying duplication and loss rates, population sizes, and number of samples to understand how these parameters affect several metrics.

**Fig. 6:**
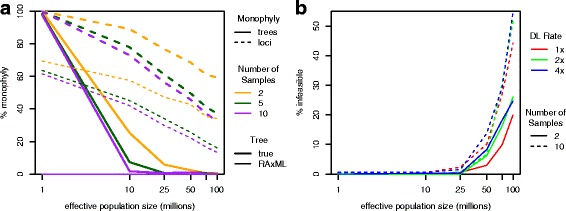
Reconciliation infeasibility in simulated flies data set. **a** The percentage of monophyletic trees and loci for varying number of samples using the true simulated gene tree and the reconstructed RAxML gene tree. Monophyly decreases as the population size and number of samples increase. Results are shown for duplications and losses simulated at the same rate (1 ×) as that estimated for real data; little difference is observed when the rate is varied (not shown). **b** The percentage of gene trees with infeasible reconciliations using RAxML gene trees. The percentage of infeasible gene trees increases as the population size, duplication-loss rate, and number of samples increase. Results for 5 samples are not shown but tend to lie between the values for 2 and 10 samples for the same population size and duplication-loss rate

As before, we investigated the monophyly of genes sampled at the same species and locus (Fig. 6 a), and as expected, we find that, as the population size and number of samples increase, each of which increases the level of ILS, monophyly decreases. Furthermore, for reconstructed gene trees, no tree is monophyletic and few loci are monophyletic, demonstrating that reconstructed gene trees exhibit greater disagreement among samples compared to true trees.

We also find that the percentage of infeasible gene trees increases with the level of ILS (Fig. 6 b). However, possible sources of ILS affect infeasibility in different ways. For example, few gene trees (0–2.3%) are infeasible for low population sizes (1–25 million), but once the population size exceeds this threshold, the percentage of infeasible gene trees increases rapidly. In contrast, increasing the rate of duplications and losses or increasing the number of samples also increases the percentage of infeasible gene trees but to a lesser degree. For example, at a duplication-loss rate 1× the estimated real rate, a population size of 50 million, and with 2 samples per species, 3.0% of gene trees are infeasible. Doubling the population size incurs a larger increase in infeasibility (17.0 percentage points to 20.0%) than increasing the number of samples to 5 (5.9 points to 8.9%) or doubling the duplication-loss rate (3.2 points to 6.2%). Interestingly, these results can only partially be attributed to trends in monophyletic loci (Fig. 6 a). That is, from the same baseline of 1×, 50 million, and 2 samples, 42.7% of loci are monophyletic. Just as doubling the duplication-loss rate yields the smallest increase in infeasibility, it also incurs the smallest decrease in monophyletic loci (0.8 points to 41.9%). However, increasing the number of samples yields a larger decrease (17.1 points to 25.6%) than doubling the population size (7.3 points to 35.4%). Still, together, these results demonstrate that the problem of infeasible gene trees must be considered for dense, rapidly evolving clades, a type of data set that is likely to increase as sequencing costs decline.

## Conclusion

Traditionally, researchers have investigated eukaryotic gene families using the duplication-loss model only or the coalescent-only model only. However, while the duplication-loss model can be applied to paralogous families with multiple loci per species, it cannot capture population-related effects and is restricted to a single sample per species. Similarly, while the coalescent model can incorporate multiple alleles and thus multiple samples per species, it assumes orthologous loci with a single locus per species. This work bridges these models by considering a joint DLC model and allowing for multiple loci *and* multiple samples per species. Importantly, only by using a joint model can we account for both sources of gene multiplicity within a species.

However, for gene families with multiple loci and samples, we have demonstrated that gene tree topological feasibility is no longer guaranteed, and to address this issue, we have presented an algorithm for assessing feasibility. Because we have allowed for data sets with multiple loci and samples, our method will only become increasingly relevant as more genomes as sequenced. For such data sets, we envision our method as part of a larger phylogenomic pipeline, for example, to identify gene trees with known errors or gene trees that violate our model assumptions and filter them from analysis. Additionally, because our method relies only on the gene tree topology and leaf mappings, and in particular, is independent of the species tree and the gene tree rooting, it is broadly applicable. In this way, our method complements existing bootstrap methods for measuring gene tree quality. While bootstraps can be used to evaluate the robustness of a reconstructed topology, both at the resolution of the full topology and for individual branches, our method can definitively identify when a gene tree topology has been affected by reconstruction error or gene conversion. However, we caution that our approach can only identify a portion of the trees that are incorrect, and the sensitivity of our method for identifying topological error remains an open question.

Going forward, our work moves us one step closer to phylogenomic studies with multiple samples per species, and we see several directions for future work. For example, we have addressed the question of reconciliation feasibility for binary gene trees. Next steps could consider feasibility for non-binary gene trees or extend current DLC-reconciliation algorithms such as DLCoalRecon [36] and DLCpar [37] to handle multiple samples per species. There has also been recent work on whether ortholog and paralog constraints, possibly inferred from external sources, are mutually satisfiable [50] and on correcting gene tree topological errors based on ortholog constraints [51]. Along these lines, our work could be extended to capture ortholog constraints so that researchers could, for example, incorporate evidence from manually-curated comparisons between model organisms to improve inferences. Or, given that we know which gene trees must have errors, one could investigate error-correction algorithms for making infeasible gene trees feasible. Finally, we have assumed that, for each species, we know the locus from which each gene was sampled. For data sets without this information, a reconciliation approach could be developed to infer relationships within species in addition to between species.

## Additional file

Additional file 1 Supplementary Material. (PDF 370 kb)
