# Supplementary material for: Reconciliation feasibility in the presence of gene duplication, loss, and coalescence with multiple individuals per species
Source: BMC Bioinformatics. 2017 Jun 5;18:292. doi: 10.1186/s12859-017-1701-1 (PMC5460407; doi:10.1186/s12859-017-1701-1)
Supplement: Supplementary file 1 — Supplementary Material. (PDF 370 kb) [file 12859_2017_1701_MOESM1_ESM.pdf]

Reconciliation Feasibility in the Presence of Gene Duplication, Loss, and Coalescence with Multiple Individuals per Species – Supplementary Material

Jennifer Rogers, Andrew Fishberg, Nora Youngs, Yi-Chieh Wu

Supplemental Methods

S1 Algorithm

We start with some basic tree and graph definitions. Let $T = (V(T), E(T))$ be an unrooted, full binary tree with a set $V(T)$ of nodes (or vertices) and a set $E(T)$ of branches (or edges). Let $L(T) \subset V(T)$ denote the set of leaves, and for nodes u and v, let $path(u, v)$ denote the set of branches along the unique simple path from u to v in T. Similarly, let $G = (V(G), E(G))$ be an undirected graph with a set $V(G)$ of vertices and a set $E(G)$ of edges. Let $\mathcal{C}(G)$ denote the set of connected components of G, where $C \in \mathcal{C}(G)$ is a subgraph of G denoting a single connected component.

Next, we define some locus terminology. Given a gene family, let locus set $\mathbb{L}$ be the set of species-specific loci that have evolved within the gene family. In general, the relationship between loci in different species is unknown. For locus l, let $s(l)$ denote the species to which l belongs.

Definition S1.1 (Locus Relationships)**.** Consider the locus set $\mathbb{L}$ for a locus tree, and let $l_1 \in \mathbb{L}$ and $l_2 \in \mathbb{L}$ be two loci such that $l_1 \neq l_2$. l_1 and l_2 are *orthologous* if their most recent common ancestor (MRCA) corresponds to a speciation event, *equivalent* if they derived from their MRCA by speciation events alone, and *reconcilable* if they could potentially be orthologous (Figure S3).

Remark. Locus equivalency is a transitive relationship, but locus orthology is not. Furthermore, equivalent loci are necessarily orthologous, but orthologous loci are not necessarily equivalent.

Remark. Absent other evidence, two loci from different species are reconcilable (and thus could be orthologous although they do not have to be). By contrast, two (different) loci from the same species are not reconcilable.

Because multiple species-specific loci may be equivalent and thus correspond to the same evolutionary locus, we introduce the concept of a *locus class*.

Definition S1.2 (Locus Class)**.** Let a collection $\mathbb{LC} = \{C_i\}$ of nonempty sets form a partition over $\mathbb{L}$ such that each locus $l \in \mathbb{L}$ belongs to a single *locus class* $C_i \in \mathbb{LC}$.

In this work, we are given as input a gene tree topology G and a leaf mapping $Le\colon L(G) \to \mathbb{L}$. That is, for each gene, we know the species-specific locus from which it was sampled and thus also the species in which it was sampled. Because we have sampled multiple individuals per species, multiple $g \in L(G)$ may map to the same $l \in \mathbb{L}$. Note that we also typically know the individual from which the gene was sampled, but this information is not used explicitly. Our goal is to determine whether G is reconcilable.

Definition S1.3 (Reconcilable Gene Tree)**.** Given a gene tree G and a leaf mapping Le, for each $g_i \in L(G)$, let $Le(g_i) = l_i$ denote the locus associated with g_i. Then, G is *reconcilable* if there exists some mapping $\mathcal{L}\colon L(G) \cup E(G) \to \mathbb{LC}$ of each leaf and edge of the gene tree to a single locus class, such that for each pair $g_1 \neq g_2$, $\mathcal{L}$ is subject to the following constraints:

1. If $l_1 = l_2$, then $\mathcal{L}(g_1) = \mathcal{L}(g_2)$ and for each $e \in path(g_1, g_2)$, $\mathcal{L}(e) = \mathcal{L}(g_1)$. (Allele Constraint)
2. If l_1 and l_2 are irreconcilable, then $\mathcal{L}(g_1) \neq \mathcal{L}(g_2)$. (Paralog Constraint)

Constraint 1 ensures that genes from the same (species-specific) locus are assigned the same locus class and that the genes and edges assigned the same locus class form a subtree of the gene tree. This latter constraint follows from our assumption that each duplication creates a unique new locus. Without this assumption, two leaves could map to the same locus if, for example, along the path from their MRCA to one of the leaves, there was a duplication to a new locus followed by a duplication back to the original locus. Constraint 2 ensures that genes from irreconcilable loci are not assigned the same locus class. That is, paralogous genes by definition cannot be orthologous, so they must be in different locus classes.

Problem S1.1 (Reconciliation Feasibility)**.** Given G and Le, determine whether G is reconcilable.

To solve Problem S1.1, we rely on two new data structures:

Definition S1.4 (Partially Labeled Coalescent Tree)**.** Let $\mathbb{P}(\mathbb{L})$ denote the power set of $\mathbb{L}$. Given G and Le, the *partially labeled coalescent tree (PLCT)* is a mapping $\mathcal{P} : E(G) \to \mathbb{P}(\mathbb{L})$ that labels branches with the species-specific locus or loci to which the branch must belong. That is, for each $e \in E(G)$, $\mathcal{P}(e)$ contains l if and only if there exists a pair of genes mapped to l and e lies along the path between those genes.

Remark. The PLCT can be thought of as a partial locus map that labels each gene tree branch with the locus in which it evolved. It is a *partial* map for two reasons: (1) There can exist gene tree branches without any label as branches are labeled only if they lie along the path between two alleles. (2) Branches are labeled with species-specific loci, but multiple species-specific loci may be equivalent and correspond to the same evolutionary locus.

Definition S1.5 (Locus Equivalence Graph)**.** Given a PLCT $\mathcal{P}$ for G and Le, the *locus equivalence graph (LEG)* is a graph $\mathcal{G}$ where $V(\mathcal{G}) = \mathbb{L}$ and for each pair $l_1 \in \mathbb{L}, l_2 \in \mathbb{L}$ such that $l_1 \neq l_2$, $E(\mathcal{G})$ contains (l_1, l_2) if and only if there exists an edge in the gene tree that is labeled with both l_1 and l_2 in the PLCT.

That is, because the locus classes are defined over the species-specific loci, the PLCT captures the allele constraints for each species-specific locus. These constraints are then put together in the LEG. In particular, if an edge of the gene tree is labeled with multiple loci in the PLCT, then the multiple loci must correspond to the same locus class and be equivalent. This equivalency constraint is captured in the LEG: the LEG contains an edge between two loci if the loci are equivalent. Note, however, that the LEG captures the loci pairs that must be equivalent, but these are not necessarily the only pairs that can be equivalent.

Next, we define the concept of a reconcilable LEG and relate it to gene tree reconcilability.

Definition S1.6 (Reconcilable Connected Component)**.** A connected component C of the locus equivalence graph $\mathcal{G}$ is *reconcilable* if and only if all of the loci in that component are pairwise reconcilable.

Definition S1.7 (Reconcilable Locus Equivalence Graph)**.** A locus equivalence graph $\mathcal{G}$ is *reconcilable* if and only if all of its connected components are reconcilable.

Theorem S1.1. *A gene tree is reconcilable if and only if its locus equivalence graph is reconcilable.*

In other words, Theorem S1.1 states that that a gene tree is reconcilable if and only if every connected component of the LEG contains no more than one locus from any species. Algorithm S1 summarizes how to generate the PLCT and LEG and determine feasibility. We can then analyze the time complexity of our algorithm.

Theorem S1.2. *Gene tree reconcilability can be decided in a total time complexity of* $O(|L(G)|^3)$.

S1.1 Proofs

Lemma S1.3. *If v is an internal node of the gene tree, and some branch incident with v has label l in the PLCT, then some other branch incident with v must also have label l.*

Proof. Suppose some branch incident with v is labeled l. Then, by the method used to label branches of the PLCT in Algorithm S1, we know that v must lie on the path between two genes from the same species-specific locus. The path between two genes must start and end at leaves of the gene tree. Therefore, since v is an internal node of the gene tree, it must have two neighbors on this path, and it must have at least two incident branches labeled l. □

Algorithm S1 DETERMINING FEASIBILITY

Input: G, Le
Output: reconciliation feasibility (**true**/**false**)

{Generating the PLCT $\mathcal{P}$ (see also footnote 1)}
for each edge $e \in E(G)$ **do**
  Initialize $\mathcal{P}(e)$ to $\varnothing$.
for each pair of nodes $g_1 \in L(G)$ and $g_2 \in L(G)$ such that $g_1 \neq g_2$ and $Le(g_1) = Le(g_2)$ **do**
  for each edge $e \in path(g_1, g_2)$ **do**
    Add locus $Le(g_1)$ to $\mathcal{P}(e)$.
{Generating the LEG $\mathcal{G}$}
Initialize $\mathcal{G}$ to $(\varnothing, \varnothing)$.
for each locus $l \in \mathbb{L}$ **do**
  Add node l to $V(\mathcal{G})$.
for each edge $e \in E(G)$ **do**
  for each pair of loci $l_1 \in \mathcal{P}(e)$ and $l_2 \in \mathcal{P}(e)$ such that $l_1 \neq l_2$ **do**
    Add edge (l_1, l_2) to $E(\mathcal{G})$.
{Determining Reconciliation Feasibility}
for each $C \in \mathcal{C}(\mathcal{G})$ **do**
  for each pair of loci $l_1 \in C$ and $l_2 \in C$ such that $l_1 \neq l_2$ **do**
    if $s(l_1) = s(l_2)$ **then**
      Return **false**.
Return **true**.

[1] For locus $l \in \mathcal{L}$, let $A(l)$ denote the set of genes mapped to l; that is, $A(l)$ is a set of alleles. Then, if $|A(l)| > 2$, rather than consider each pair of genes, we can equivalently find the subtree of G that spans $A(l)$ and for each edge e in this subtree, add l to $\mathcal{P}(e)$.

Lemma S1.4. *Suppose C_1 and C_2 are maximal disjoint connected components of the locus equivalence graph. Next, suppose that $g_1 \in L(G)$ is a gene from locus $l_1 \in C_1$ and $g_2 \in L(G)$ is a gene from locus $l_2 \in C_2$. Then, there exists an edge between g_1 and g_2 of the gene tree that is unlabeled in the PLCT.*

Proof. Note that $l_1 = Le(g_1)$ and $l_2 = Le(g_2)$ are the labels associated with the loci of g_1 and g_2, respectively. Assume, by way of contradiction, that there is no unlabeled edge on the path p from g_1 to g_2.

Now consider the traversal of p from g_1 to g_2. The first edge in this traversal must be labeled l_1. To see this, note that every edge of p is labeled with some l_i. By our definition of the PLCT, edges are only labeled when they lie on the path between two genes with the same species-specific locus. Since the first edge in p is incident to a leaf sampled from l_1, it is impossible to label the first branch of p anything but l_1. The same argument shows that the last edge in p must be labeled l_2.

Thus, p begins with label l_1 and ends with label l_2. Then, if we traverse p from g_1 to g_2, there must be some "first edge" e_f labeled l_i, where l_i and l_1 are from different connected components of the LEG. We know this is true because l_1 and l_2 are from disjoint connected components of the LEG.

Let v be the vertex immediately preceding edge e_f in p. We know that v is incident with an edge with label l_1 and with an edge with label l_i. Then, by Lemma S1.3, v must be incident with at least two edges with label l_1 and with at least two edges with label l_i. However, since v is an internal vertex of a binary tree, it has degree 3. Therefore, some edge incident with v must be labeled both l_1 and l_i, and thus, l_1 and l_i must be connected in the LEG. This is a contradiction because we claimed that l_1 and l_i were loci from different connected components of the LEG.

We have reached a contradiction, so there must be some unlabeled edge on the path p from g_1 to g_2. □

Lemma S1.5. *A gene tree is reconcilable if and only if the following conditions hold:*

N1. There exists no duplication along the path between two genes from the same locus. (Allele Constraint)

N2. There exists a duplication along the path between two genes from irreconcilable loci. (Paralog Constraint)

Proof. We will show that these criteria are equivalent to our original criteria for a reconcilable gene tree, which are repeated here:

1. Genes from the same locus must be assigned the same locus class, and all edges along the path between these genes must be assigned the same locus class as the genes. (Allele Constraint)
2. Genes from irreconcilable loci must not be assigned the same locus class. (Paralog Constraint)

Throughout this proof, note that each gene or edge of the gene tree is assigned a single locus class. Most importantly, we observe that a duplication along a path must result in a change of locus class; moreover, this is the only way for a branch to change locus class, and the change is not reversible – a subsequent duplication along the path cannot revert back to a previously used locus class. Thus, Constraint 1 and Constraint N1 are equivalent, as genes from the same locus are assigned the same locus class along with all the edges between them precisely when there is no duplication along the path. Similarly, Constraint 2 and Constraint N2 are equivalent since genes from irreconcilable loci are assigned different locus classes if and only if there is a duplication somewhere on the path between the two. □

Proof of Theorem S1.1

Suppose the locus equivalence graph is reconcilable. By Lemma S1.5, we have a definition for a reconcilable gene tree in terms of duplications, the constraints of which are repeated here:

N1. There exists no duplication along the path between two genes from the same locus.

N2. There exists a duplication along the path between two genes from irreconcilable loci.

That is, if we have a procedure to add duplications to our gene tree such that these constraints hold, then we have demonstrated that the gene tree is reconcilable. Consider the following procedure. For each pair of genes $g_1 \in L(G)$, $g_2 \in L(G)$ such that $g_1 \neq g_2$ and $l_1 = Le(g_1)$ and $l_2 = Le(g_2)$ are irreconcilable, consider the path from g_1 to g_2. Since the LEG is reconcilable, two irreconcilable loci must be found in different connected components of the LEG, so by Lemma S1.4, there is an unlabeled edge on this path. Add a duplication on some unlabeled edge. Now, we have satisfied Constraint N2. We also claim that we have satisfied Constraint N1 because we never added a duplication on an edge that was labeled by the PLCT; therefore, we never added a duplication on the path between two genes from the same locus.

For the converse, we will prove the contrapositive: if the locus equivalence graph is irreconcilable, then the gene tree is also irreconcilable. Suppose the LEG is irreconcilable, and assume, by way of contradiction, that the gene tree is reconcilable using our original definition for a reconcilable gene tree, the constraints of which are repeated here:

1. Genes from the same locus must be assigned the same locus class, and all edges along the path between these genes must be assigned the same locus class as the genes.
2. Genes from irreconcilable loci must not be assigned the same locus class.

Throughout the remainder of this proof, we say that a (species-specific) locus is assigned a locus class if all genes associated with that locus are assigned the locus class, and similarly, two (species-specific) loci are assigned different locus classes if all pairs of genes, one from each loci, are assigned different locus classes.

Since the LEG is irreconcilable, there must be some connected component that is irreconcilable and this connected component must contain some pair of loci l_1 and l_2 that are irreconcilable. Thus, by Constraint 2, l_1 and l_2 must not be assigned the same locus class. Next, recall that the LEG contains an edge between two loci if the loci are equivalent. Since locus equivalency is transitive, any two loci in a given connected component must be equivalent. So, since l_1 and l_2 are in the same connected component, they must be equivalent (and correspond to the same evolutionary locus) and, by Constraint 1, be assigned the same locus class. We have reached a contradiction in that loci l_1 and l_2 must be assigned and not be assigned the same locus class, so the gene tree must be irreconcilable. □

Remark. The proof for the converse does not rely on any lemmas and, in particular, does not require a binary gene tree. Thus, for a *non-binary* gene tree, it holds that if the LEG is irreconcilable, the gene tree is irreconcilable. But, it does *not* hold that if the LEG is reconcilable, the gene tree is reconcilable.

Remark. If a reconcilable gene tree exists, this proof yields a procedure for generating a valid labeling of the gene tree, that is, a labeling that satisfies Definition S1.3. We consider all pairs of irreconcilable loci and for each, insert a duplication on an unlabeled edge between the two loci. Next, we cut the tree at the duplications and assign each leaf and edge within the same subgraph to a single locus class. As stated in the proof, this necessarily satisfies the definition of a reconcilable gene tree, thus, yielding a valid labeling. Because there may be multiple ways to insert duplications according to this procedure, multiple valid labelings may exist.

Proof of Theorem S1.2

We consider the time complexity for each subroutine of Algorithm S1 line-by-line. Let $n = |L(G)|$ and $k = |\mathbb{L}|$. We assume that $\mathbb{L}$ is represented using non-negative integers and that the mapping of loci to species is implemented using an array of size k so that for $l \in \mathbb{L}$, $s(l)$ requires $O(1)$ time. Further, for each $e \in E(G)$, $\mathcal{P}(e)$ is implemented as a boolean array of size k using one-hot encoding: for each $l \in \mathbb{L}$, index l of $\mathcal{P}(e)$ is `TRUE` if $\mathcal{P}(e)$ contains l and is `FALSE` otherwise. So, initializing $\mathcal{P}(e)$ to $\varnothing$ requires $O(k)$ time and adding an element to $\mathcal{P}(e)$ requires $O(1)$ time. Additionally, we assume that $\mathcal{G}$ is implemented as an adjacency matrix so that adding vertices and edges requires $O(1)$ time.

The initialization at lines 1–2 requires $O(nk)$ time. The outer 'for' loop at line 3 is executed $O(n^2)$ times. Determining the path between two nodes requires $O(n)$ time, so the total complexity for all paths in line 4 in $O(n^3)$. Furthermore, the inner 'for' loop at line 4 is executed $O(n)$ times, and since line 5 requires $O(1)$ time, the total complexity of line 5 is $O(n^3)$. Thus, the PLCT subroutine requires $O(nk + n^3)$ time.

The graph initialization at line 6 requires $O(1)$ time, and the next 'for' loop at lines 7–8 requires $O(k)$ time. The 'for' loops at lines 9 and 10 are executed $O(n)$ and $O(k^2)$ times, respectively, giving a total complexity of $O(nk^2)$ for line 11. Thus, the LEG subroutine requires $O(nk^2)$ time.

Determining connected components requires $O(|V(\mathcal{G})| + |E(\mathcal{G})|) = O(k^2)$ time using breadth-first-search or depth-first-search, where $|V(\mathcal{G})| = k$ and $|E(\mathcal{G})| \leq \frac{k(k-1)}{2}$. The outer 'for' loop at line 12 is executed $O(k)$ times since there are at most k connected components, and the inner 'for' loop at line 13 is executed $O(k^2)$ times since a single component has at most k vertices. This gives a total time complexity of $O(k^3)$ for line 15. Thus, the feasibility subroutine requires $O(k^3)$ time.

Put together, the total time complexity of the algorithm is $O(n^3 + nk + nk^2 + k^3)$. Since $k \leq n$, the complexity can alternatively be written as $O(n^3)$.

S1.2 Optimization

The approach for generating the PLCT in Algorithm S1 can be decreased from a total time complexity of $O(n^3)$ to a complexity of $O(nk)$, where $k = |\mathbb{L}|$. We accomplish this optimization by rooting the gene tree arbitrarily along any branch, allowing us to traverse it in preorder and postorder.

Throughout this section, let $T = (V(T), E(T))$ be a rooted, full binary tree with a set $V(T)$ of nodes and a set $E(T)$ of directed branches. Let $L(T) \subset V(T)$ be the set of leaves, $I(T) = V(T) \setminus L(T)$ be the set of internal nodes, and $r(T) \in I(T)$ be the root node. For node v, let $\mathit{left}(v)$ be its left child, $\mathit{right}(v)$ be its right child, $\mathit{parent}(v)$ be its parent, and $T(v)$ be the (maximal) subtree of T rooted at v.

Additionally, we define a modified PLCT $\mathcal{P}' \colon V(G) \to \mathbb{P}(\mathbb{L})$ that labels each gene tree *node* with the species-specific locus or loci to which the node must belong. That is, for each $g \in L(G)$, $\mathcal{P}'(g)$ contains $Le(g)$, and for each $g \in I(G)$, $\mathcal{P}'(g)$ contains l if and only if there exists a pair of genes mapped to l and g lies along the path between those genes. Then, for each $e = (u, v) \in E(G)$, the original PLCT can be defined as $\mathcal{P}(e) = \mathcal{P}'(u) \cap \mathcal{P}'(v)$; that is, an edge is labeled with l if the path goes through both vertices of the edge.

Algorithm S2 summarizes the optimized algorithm for generating the PLCT. Note that substituting Algorithm S2 for the PLCT component of Algorithm S1 (lines 1-5) reduces the total time complexity for Algorithm S1 from $O(n^3)$ to $O(nk + nk^2 + k^3) = O(nk^2 + k^3)$, which is in general faster because $k \leq n$.

Proof of Correctness

In the postorder traversal, for each gene $g \in V(G)$, we compute set $\mathcal{U}(g)$, which, for leaves, contains the species-specific locus of g (line 4), and for internal nodes, contains the labels that appear in any leaf descended from g (line 7). Next, for each leaf $g \in L(G)$, $\mathcal{P}'(g)$ contains only $Le(g)$ (line 5). For each internal node $g \in I(G)$, if $\mathcal{U}(\mathit{left}(g))$ and $\mathcal{U}(\mathit{right}(g))$ contain the same label l, then there must exist a pair of genes $g_1 \in L(T(\mathit{left}(g)))$ and $g_2 \in L(T(\mathit{right}(g)))$ [that is, g_1 and g_2 are in the set of leaves in the left and right subtrees of g, respectively] such that g_1 and g_2 map to the same locus l. Thus, there exists a path between g_1 and g_2, both mapped to l, that goes through g, and so $\mathcal{P}'(g)$ must contain l (line 8). So, at the end of the postorder traversal, for each $g \in L(G)$, $\mathcal{P}'(g)$ is computed correctly, and for each $g \in I(G)$, $\mathcal{P}'(g)$ contains l if and only if there exists a pair of genes *in the subtree of G rooted at g* such that both genes map to l and g lies along the path between those genes. Note that since $T(r(G)) = G$, it must be that $\mathcal{P}'(r(G))$ is computed correctly.

Algorithm S2 PLCT OPTIMIZATION

```
Input: G, Le
Output: 𝒫
    {Generating the PLCT}
 1: Root G arbitrarily.
 2: for each node g ∈ V(G) in postorder do
 3:   if g ∈ L(G) then
 4:     Set 𝒰(g) = {Le(g)}.
 5:     Set 𝒫′(g) = {Le(g)}.
 6:   else
 7:     Set 𝒰(g) = 𝒰(left(g)) ∪ 𝒰(right(g)).
 8:     Set 𝒫′(g) = 𝒰(left(g)) ∩ 𝒰(right(g)).
 9: for each node g ∈ V(G) in preorder do
10:   if g ∈ I(G) \ {r(G)} then
11:     Update 𝒫′(g) = 𝒫′(g) ∪ (𝒫′(parent(g)) ∩ 𝒰(g)).
```

Then, for each internal node g that is not the root, what remains is to update $\mathcal{P}'(g)$ with the labels induced by pairs of genes, one in the subtree of G rooted at g and one in the rest of G, such that both genes map to l. We preorder traverse the gene tree to perform this update and prove by induction that we correctly update $\mathcal{P}'(g)$. For the base case, observe that $\mathcal{P}'(r(G))$ is correct. Assume, without loss of generality, that we are updating $\mathcal{P}'(g)$ where $g = \mathit{left}(r(G))$. Recall that $\mathcal{U}(g)$ contains labels that appear in any leaf descended from g. At the same time, $\mathcal{P}'(\mathit{parent}(g))$ contains labels that must be "propagated" down the tree. So, if $\mathcal{U}(g)$ and $\mathcal{P}'(\mathit{parent}(g))$ contain the same label l, then there must exist a pair of genes $g_1 \in L(T(g))$ and $g_2 \in L(G) \setminus L(T(g))$ such that g_1 and g_2 map to the same locus l. And, as before, there exists a path between g_1 and g_2, both mapped to l that goes through g, and so $\mathcal{P}'(g)$ must contain l (line 11). Thus, the sets $\mathcal{P}'(g)$ for each $g = \mathit{left}(r(G))$ and $g = \mathit{right}(r(G))$ are computed correctly. Induction completes our proof. □

Proof of Complexity

We consider the time complexity for each component of Algorithm S2 line-by-line. As before, we assume that, for each $g \in V(G)$, $\mathcal{U}(g)$ and $\mathcal{P}'(g)$ are implemented using arrays of size l with one-hot encoding. Thus, the union and intersection of two sets requires $O(k)$ time.

Rooting a tree requires $O(n)$ time. The first 'for' loop at line 2 is executed $O(n)$ times, and lines 4–5 each require $O(1)$ time and lines 7–8 each $O(k)$ time, giving a total time complexity of $O(nk)$ for lines 3–8. The second 'for' loop at line 9 is also executed $O(n)$ times, and line 11 requires $O(k)$ time, giving a total time complexity of $O(nk)$ for line 11. Thus, the total time complexity of the algorithm is $O(nk)$. □

S2 Biological Gene Trees

We analyzed seven species or subspecies within the great apes clade: humans (*Homo sapiens*), Western chimpanzees (*Pan troglodytes verus*), bonobos (*Pan paniscus*), Eastern lowland gorilla (*Gorilla beringei graueri*), Western lowland gorilla (*Gorilla gorilla gorilla*), Sumatran orangutans (*Pongo abelii*), and Bornean organutans (*Pongo pygmaeus*). We obtained reference genomes and variants from Prado-Martinez et al. (2013) and genome annotations from Ensembl release 72 (Flicek et al. 2014). For species without a reference genome (bonobos and Bornean orangutans), we used as reference the genome of its closest relative (chimpanzees and Sumatran orangutans). For the non-human species, we retained only variants that passed all filters using VCFtools (Danecek et al. 2011), imputed and phased genotypes using Beagle 4.0 (Browning and Browning 2007), and extracted autosomal variants in protein-coding regions and applied them to the reference fasta sequences using VCFtools. For humans, we performed a similar procedure except that we imputed and phased genotypes against the 1000 Genomes Project (The 1000 Genomes Project Consortium 2012). To reduce the number of samples and proteins under consideration, we retained the two highest-depth samples from each species and analyzed the longest protein sequence per gene.

Next, we obtained 10,800 autosomal gene family definitions from Ensembl release 72 (Flicek et al. 2014) and retained 7988 families that contained a variant in at least one gene within the family, yielding 15,976 families across two haplotypes. Of these, we retained 6298 "non-trivial" families that contained at least two loci from any one species. We aligned protein sequences using MUSCLE (Edgar 2004), reverse translated the protein alignment to a (codon-aligned) nucleotide alignment, and reconstructed gene trees using four programs: PHYLIP (Felsenstein 1989), BioNJ (Gascuel 1997), PhyML (Guindon et al. 2010) with the GTR model, and RAxML (Stamatakis 2006) with 100 fast bootstraps and the GTRGAMMA model.

S3 Simulated Gene Trees

In Rasmussen and Kellis (2012), the authors used the species tree of the *Drosophila* 12 Genomes Consortium (2007) with estimated divergence times (Tamura et al. 2004), gene duplication and loss rates of 0.0012 events/gene/million years (Hahn et al. 2007), and a generation time of 10 generations/year (Sawyer and Hartl 1992, Pollard et al. 2006). While *Drosophila melanogaster* is estimated to have an effective population size N_e of $\sim$1.15 million individuals (Charlesworth 2009), they used a wide range of population sizes and also a range of duplication-loss rates to investigate the effects of varying levels of gene tree-species tree incongruence, with each set of parameters used to simulate 500 gene families. For each parameter setting, we retained only the "non-trivial" families that contained at least two loci from any one species.

As our algorithm relies on multiple samples per species, we used the locus trees of Rasmussen and Kellis (2012) and, for each locus tree, simulated gene trees with N samples per species for $N = 2, 5, 10$. These gene trees are necessarily reconcilable; that is, they have no topological error that could result in infeasible reconciliations. Thus, we simulated alignments of 1000 nucleotides for each gene tree under a HKY model (Hasegawa et al. 1985) and with a substitution rate of 5×10^{-9} substitutions/site/generation (Kimura 1968, Haag-Liautard et al. 2007) using seq-gen (Rambaut and Grassly 1997), and finally, we reconstructed gene trees from these alignments using RAxML (Stamatakis 2006) with 100 fast bootstraps and the GTRGAMMA model.

Note that, in line with our model assumptions, our simulation procedure does not allow for events such as horizontal gene transfer or gene conversion. Those interested in this more general model may consider SimPhy (Mallo et al. 2016).

Supplemental Figures

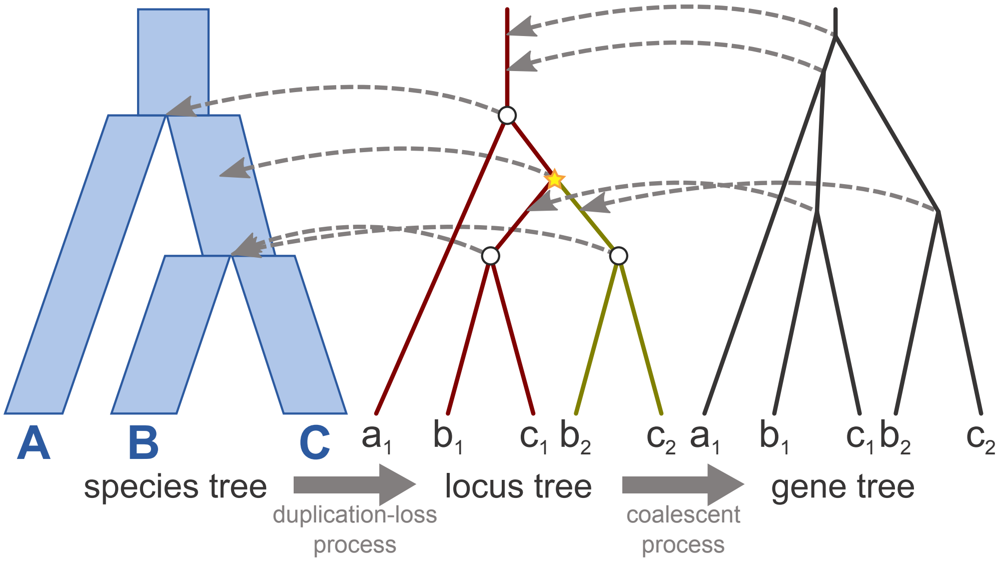

Fig S1. Generative process for the DLCoal model. Given a species tree with known topology and divergence times, a top-down duplication-loss process generates a locus tree. Branches that evolve in the daughter locus (rather than in the mother locus) of a duplication are denoted in a separate color. From the locus tree, a bottom-up coalescent process generates a gene tree. Mappings between the trees indicate how one tree “fits inside” the other. This diagram depicts the same gene evolutionary history as Figure 1c-d. [Figure and caption adapted with permission from Wu et al. (2014) and Rasmussen and Kellis (2012).]

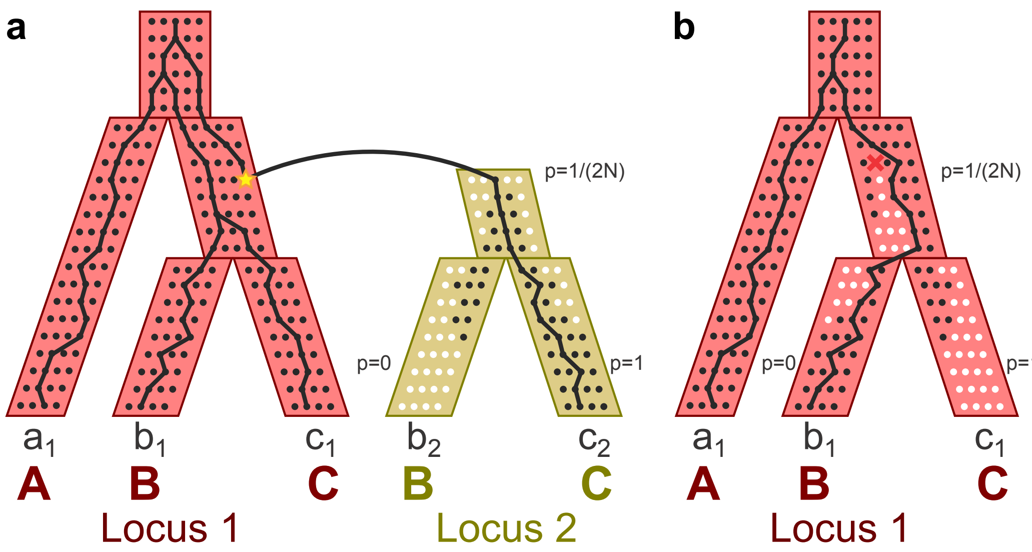

Fig S2. Duplication and loss hemiplasy. A new (**a**) gene duplicate or (**b**) gene loss can undergo hemiplasy, in which the mutation fixes in some lineages and goes extinct in others. [Parts of this figure and caption adapted with permission from Rasmussen and Kellis (2012).]

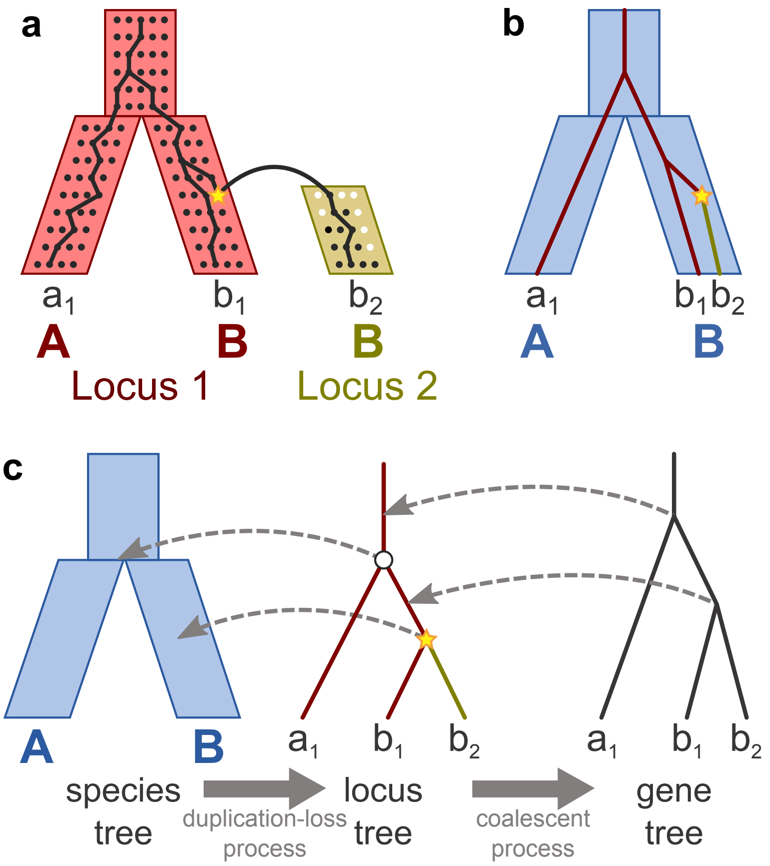

Fig S3. Locus relationships. Evolution of a gene family is shown using (**a**) the unified model, (**b**) the three-tree model, and (**c**) the LCT.

1. A locus set $\mathbb{L}$ contains the set of species-specific loci that have evolved within the gene family. In this example, $\mathbb{L} = \{a_1, b_1, b_2\}$, where species A has locus $\{a_1\}$ and species B has loci $\{b_1, b_2\}$ such that the relationship between loci in different species is unknown.
2. Two loci are orthologous if their MRCA corresponds to a speciation event (white circle in the locus tree). For the depicted history, loci a_1 and b_1 and loci a_1 and b_2 are orthologous.
3. Two loci are equivalent if they derived from their MRCA by speciation events alone. For the depicted history, loci a_1 and b_1 are equivalent. Note that loci a_1 and b_2 are orthologous but not equivalent because a duplication (yellow star) created a new locus ("locus 2") distinct from the original locus ("locus 1").
4. Two loci are reconcilable if they could potentially be orthologous. In this example, loci a_1 and b_1 and loci a_1 and b_2 are reconcilable. The depicted history shows how a_1 and b_1 would be orthologous. For a different history in which b_1 evolved in the new locus and b_2 in the original locus, a_1 and b_2 would be orthologous. In contrast, loci b_1 and b_2 from the same species must be paralogs; thus, they cannot be orthologous and are not reconcilable.

Supplemental Tables

Table S1. Bootstrap support.

conflict type	branch type	sample size	median	mean	Mann-Whitney U statistic	one-sided p-value
weak	non-conflicting	$62,337$	25	33.2	4.6×10^7	2.01×10^{-133}
	conflicting	$1,033$	3	14.2		
strong	non-conflicting	$62,884$	25	33.0	2.1×10^7	1.44×10^{-45}
	conflicting	486	5	17.9		

References

Browning S. R and Browning B. L. 2007. Rapid and accurate haplotype phasing and missing-data inference for whole-genome association studies by use of localized haplotype clustering. Am J Hum Genet **81**:1084–1097.

Charlesworth B. 2009. Fundamental concepts in genetics: Effective population size and patterns of molecular evolution and variation. Nat Rev Genet **10**:195–205.

Danecek P, Auton A, Abecasis G, Albers C. A, et al. (13 co-authors). 2011. The variant call format and VCFtools. Bioinformatics **27**:2156–2158.

Edgar R. 2004. MUSCLE: multiple sequence alignment with high accuracy and high throughput. Nucleic Acids Res **32**:1792–1797.

Felsenstein J. 1989. PHYLIP - Phylogeny Inference Package (Version 3.2). Cladistics **5**:164–166.

Flicek P, Amode M. R, Barrell D, Beal K, et al. (52 co-authors). 2014. Ensembl 2014. Nucleic Acids Research **42**:D749–D755.

Gascuel O. 1997. BIONJ: an improved version of the NJ algorithm based on a simple model of sequence data. Mol Biol Evol **14**:685–695.

Guindon S, Dufayard J, Lefort V, Anisimova M, Hordijk W and Gascuel O. 2010. New algorithms and methods to estimate maximum-likelihood phylogenies: Assessing the performance of PhyML 3.0. Syst Biol **59**:307–321.

Haag-Liautard C, Dorris M, Maside X, Macaskill S, Halligan D. L, Charlesworth B and Keightley P. D. 2007. Direct estimation of per nucleotide and genomic deleterious mutation rates in *Drosophila*. Nature **445**:82–85.

Hahn M. W, Han M. V and Han S.-G. 2007. Gene family evolution across 12 *Drosophila* genomes. PLoS Genet **3**:e197–.

Hasegawa M, Kishino H and Yano T.-a. 1985. Dating of the human-ape splitting by a molecular clock of mitochondrial DNA. J Mol Evol **22**:160–174.

Kimura M. 1968. Evolutionary rate at the molecular level. Nature **217**:624–26.

Mallo D, De Oliveira Martins L and Posada D. 2016. Simphy: Phylogenomic simulation of gene, locus, and species trees. Syst Biol **65**:334–344.

Pollard D. A, Iyer V. N, Moses A. M and Eisen M. B. 2006. Widespread discordance of gene trees with species tree in *Drosophila*: Evidence for incomplete lineage sorting. PLoS Genet **2**:e173–.

Prado-Martinez J, Sudmant P. H, Kidd J. M, Li H, et al. (75 co-authors). 2013. Great ape genetic diversity and population history. Nature **499**:471–475.

Rambaut A and Grassly N. C. 1997. Seq-Gen: An application for the Monte Carlo simulation of DNA sequence evolution along phylogenetic trees. Comput Appl Biosci **13**:235–238.

Rasmussen M. D and Kellis M. 2012. Unified modeling of gene duplication, loss, and coalescence using a locus tree. Genome Res **22**:755–765.

Sawyer S. A and Hartl D. L. 1992. Population genetics of polymorphism and divergence. Genetics **132**:1161–1176.

Stamatakis A. 2006. RAxML-VI-HPC: maximum likelihood-based phylogenetic analyses with thousands of taxa and mixed models. Bioinformatics **22**:2688–2690.

Tamura K, Subramanian S and Kumar S. 2004. Temporal patterns of fruit fly (*Drosophila*) evolution revealed by mutation clocks. Mol Biol Evol **21**:36–44.

Drosophila 12 Genomes Consortium. 2007. Evolution of genes and genomes on the *Drosophila* phylogeny. Nature **450**:203–218.

The 1000 Genomes Project Consortium. 2012. An integrated map of genetic variation from 1,092 human genomes. Nature **491**:56–65.

Wu Y.-C, Rasmussen M. D, Bansal M. S and Kellis M. 2014. Most parsimonious reconciliation in the presence of gene duplication, loss, and deep coalescence using labeled coalescent trees. Genome Research **24**:475–486.
